# Experimental evaluation of timing and preference of surgical ıntervention for crush syndrome in disaster scenarios: fasciotomy or amputation? A rat model study

**DOI:** 10.1186/s13018-025-05927-5

**Published:** 2025-05-22

**Authors:** Şehmuz Kaya, Yunus Can Ünal, Necip Güven, Abdulrahim Dündar, Ömer Faruk Keleş, Yıldıray Başbuğan

**Affiliations:** 1 Department of Orthopaedics and Traumatology, Faculty of Medicine, Van Yüzüncü Yıl University, Van, Turkey; 2 Department of Orthopaedics and Traumatology, Van Education And Research Hospital, Van, Turkey; 3 Department of Orthopaedics and Traumatology, Faculty of Medicine, Hitit University, Corum, Turkey; 4 Department of Pathology, Faculty of Veterinary Medicine, Van Yüzüncü Yıl University, Van, Turkey; 5 Department of Internal Medicine, Faculty of Veterinary Medicine, Van Yüzüncü Yıl University, Van, Turkey

**Keywords:** Crush syndrome, Rhabdomyolysis, Earthquake, Fasciotomy, Amputation

## Abstract

**Background:**

Crush syndrome is a severe condition caused by the systemic effects of rhabdomyolysis due to prolonged muscle compression. Common in disasters like earthquakes, it poses life-threatening risks, including acute renal failure, hyperkalemia, and metabolic acidosis. Although surgical interventions such as fasciotomy and amputation are critical in its management, the optimal timing and criteria remain unclear. This study investigates the impact of surgical intervention timing on crush syndrome outcomes, providing guidance through the first experimental rat model evaluating fasciotomy and amputation post-injury.

**Methods:**

Forty-eight Wistar albino rats were divided into six groups. Rhabdomyolysis was induced experimentally, followed by amputation or fasciotomy at 0, 12, or 24 h. The control group underwent rhabdomyolysis induction without surgery. Weekly urine samples were collected during the study, and blood, muscle, and kidney tissues were examined biochemically and histopathologically at the experiment's end. Data analysis focused on the systemic and tissue-specific effects of intervention timing.

**Results:**

Early intervention groups (0 h) demonstrated minimal muscle inflammation and necrosis, preserved muscle fiber arrangement, and more pronounced regeneration. Late interventions (12 and 24 h) were associated with intense inflammation, necrosis, edema, and hemorrhage in muscle tissue. Immediate amputation (0 h) limited toxic metabolite effects, reducing kidney inflammation and damage. Late interventions showed increased interstitial nephritis and inflammatory cell infiltration in kidney tissue. Biochemical analyses revealed that urinary myoglobin levels decreased and renal function was preserved in the early intervention groups.

**Conclusions:**

The timing of surgical intervention is critical in managing crush syndrome. Early amputation and fasciotomy minimized necrosis and inflammation in muscle tissue, supported tissue regeneration, and reduced systemic complications by preventing toxic metabolite accumulation in the kidneys. Early amputation particularly showed superior renal protection and lower systemic complication risks compared to late interventions. These findings highlight the importance of timely surgical action and provide valuable insights for developing effective treatment strategies in disaster settings. However, the descriptive nature of the study and the fact that the data obtained from the experimental model cannot be directly applied to clinical practice should be taken into account. Therefore, the findings should be supported by future clinical trials.

**Supplementary Information:**

The online version contains supplementary material available at 10.1186/s13018-025-05927-5.

## Background

Crush syndrome (CS) is a condition that manifests with the systemic effects of rhabdomyolysis in the body, which is a consequence of prolonged compression of muscle tissues in the extremities. It is frequently observed in the aftermath of natural disasters, such as earthquakes [1]. Prolonged exposure of muscle tissue to pressure results in structural disruptions to the cell membrane. Consequently, substances such as myoglobin, potassium and phosphate are transported into the bloodstream. Consequently, this may result in life-threatening complications, including hyperkalaemia, metabolic acidosis and acute renal failure [2, 3]. CS is typically associated with high mortality rates and is life-threatening if necessary treatments are not administered, particularly in cases where large muscle groups are affected [4]. There is no clear consensus regarding the efficacy of surgical interventions such as fasciotomy or amputation in combating CS [5]. Fasciotomy is a surgical procedure performed with the objective of reducing the intracompartmental pressure in crush injuries, thereby protecting muscle tissue and ensuring adequate tissue perfusion [6]. Compartment syndrome may result in significantly reduced circulation in the affected extremities and irreversible tissue damage. Consequently, prompt intervention is of paramount importance [7–9]. The timing of the procedure is of great consequence in determining the success of the operation. Fasciotomy performed within the first 8–10 h after injury may prevent muscle necrosis, preserve circulation, and reduce the risk of acute renal failure. It should be noted that this time interval represents the optimal intervention window [2, 10]. The incidence of adverse effects is higher when fasciotomy is performed at a later stage [11]. In the event that a period of more than 48 h has elapsed since the initial trauma, the performance of a fasciotomy may precipitate an increased risk of infection and sepsis, which in turn elevates the probability of limb loss [2, 6, 12, 13].

Amputation is a surgical procedure employed in the advanced stages of CS, particularly when the risk of infection is elevated and tissue necrosis cannot be managed effectively [8]. The decision to perform an amputation is typically made in order to prevent a life-threatening condition from developing further. In order to make this decision, a detailed and comprehensive clinical evaluation process must be undertaken [2, 7]. The issue of amputation during natural disasters, particularly earthquakes, is one that is frequently encountered, complex and controversial.

In the Kahramanmaraş earthquake of 6 February 2023 [14], which caused over 100,000 injuries and over 50,000 deaths in our country, there was considerable uncertainty regarding the optimal timing of fasciotomy and amputation procedures to prevent CS following rhabdomyolysis. This was the primary rationale for undertaking this study. The existing literature is supported by a number of retrospective studies, particularly those conducted in the aftermath of natural disasters such as earthquakes. The irreproducible and distinctive nature of disasters necessitates that researchers investigate treatment modalities by utilizing animal models of crush injuries [15]. To the best of our knowledge, our study is the inaugural experimental rat study in the literature in which fasciotomy and amputation were performed after crush injury.

This study was designed as a descriptive study. The aim of this study is to evaluate the effects of fasciotomy and amputation on crush syndrome in an experimental model and to provide clinicians with preliminary information on the timing of these interventions. The findings of the study aim to provide a basis for further research.

## Methods

Prior to the commencement of the study, permission was obtained from the Van Yüzüncü Yıl University Experimental Animals Local Ethics Committee for the procedures to be applied to the experimental animals (Date: 01.06.2023, Decision no: 2023/07–13). A total of 48 Wistar albino rats, with an average age of three months and an average weight of 250 g, were obtained. The 48 rats were randomly divided into six groups, with eight rats in each group. Throughout the experiment, the cages were maintained in rooms with an average temperature of 22 to 24 °C and 40 to 60% humidity, with a 12-h dark/12-h light cycle. During the study, the rats were provided with unlimited access to tap water and standard rodent feed.

Group 1 (control group) In this experimental group, no intervention was conducted following the induction of rhabdomyolysis.

Group 2 (amputation group at 0 h): In this group, both hind limbs were amputated without delay following the induction of rhabdomyolysis.

Group 3 (fasciotomy performed at 0 h): In this group, a fasciotomy was conducted on both hind limbs without delay following the induction of rhabdomyolysis.

Group 4 (amputation at 12 h): In this group, the amputation of both hind limbs was performed 12 h after the induction of rhabdomyolysis.

Group 5 (fasciotomy performed at the 24 th hour): In this group, fasciotomy was performed on both hind limbs 24 h after rhabdomyolysis was induced.

Group 6 (amputation group at the 24 th hour): In this group, amputation was performed on both hind limbs 24 h after rhabdomyolysis was induced.

### Experimental system components

**Fig. 1:**
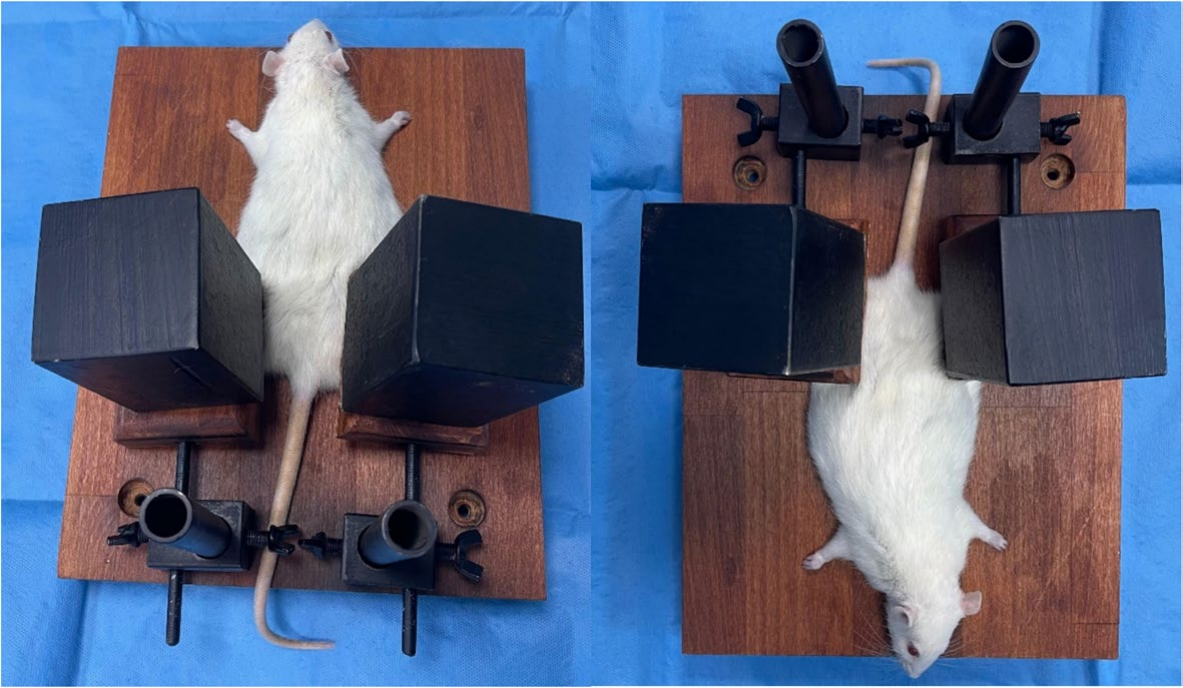
Rat in rhabdomyolysis model

A device previously described in the literature was prepared for use in creating a rat model of rhabdomyolysis injury [16]. This model represents the most accurate method to simulate injury caused by earthquakes [15]. At the centre of the apparatus is a platform on which the rat is placed. This platform was used to apply pressure to the hind limbs of the rat using boards attached to an upright bar on which 6 kg iron weights were placed (Fig. 1).

### Anaesthesia and surgical procedure

The anaesthetic regimen consisted of Xylazine (Rompun, Bayer) 10 mg/kg and Ketamine hydrochloride (Ketamine, Pfizer) 75 mg/kg, administered intraperitoneally. The rats were maintained in the rhabdomyolysis experimental system for a period of six hours.

**Fig. 2:**
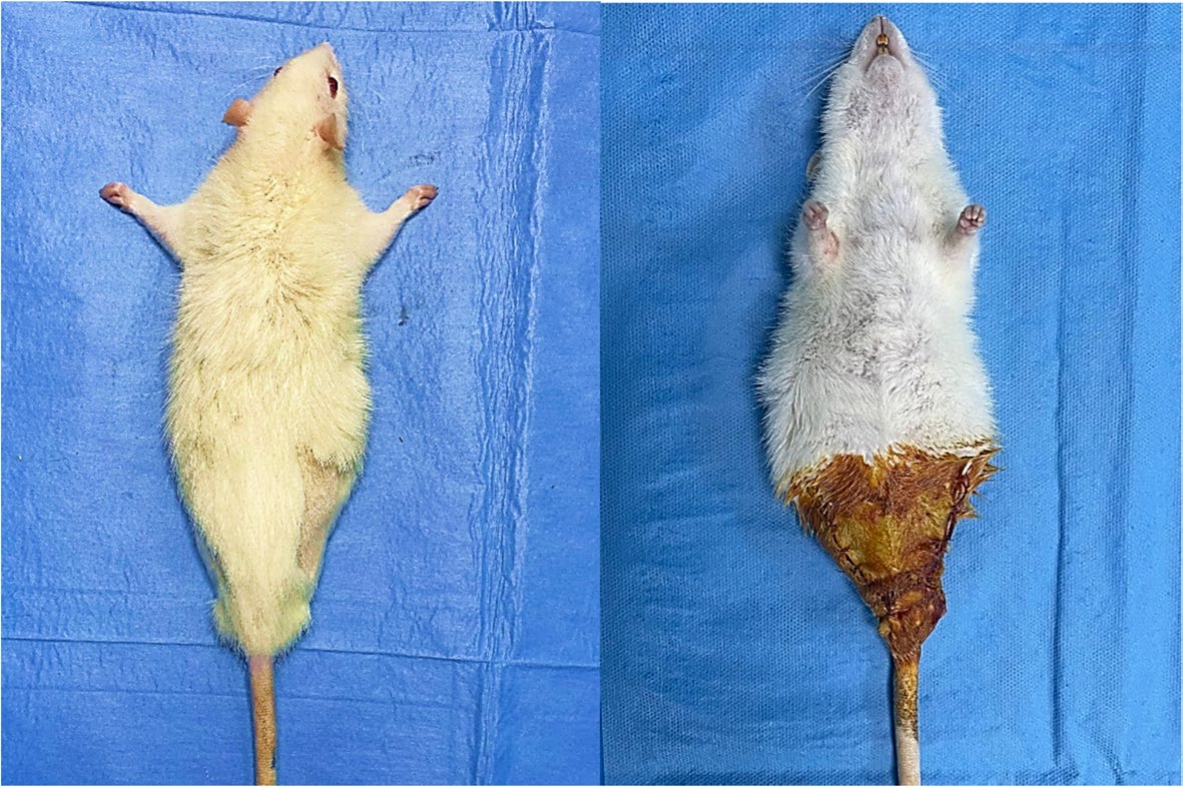
Bilateral hind limb amputated rat (**A**) postoperative 1 st week (**B**) peroperative period

Anaesthesia was reintroduced for surgical procedures, and cefazolin sodium was administered intraperitoneally in a single dose of 15 mg/kg as a prophylactic measure against surgical site infection. Then, in the amputation groups, the right and left hip regions were shaved and prepared with 10% povidone-iodine. Subsequently, the hip joint was accessed through a 2–3 cm incision in the lateral aspect of both hips, and amputation was performed (Fig. 2). This was done in a sterile manner. Once haemostasis had been achieved and the wound irrigated, the wound was closed with 3/0 absorbable sutures.

**Fig. 3:**
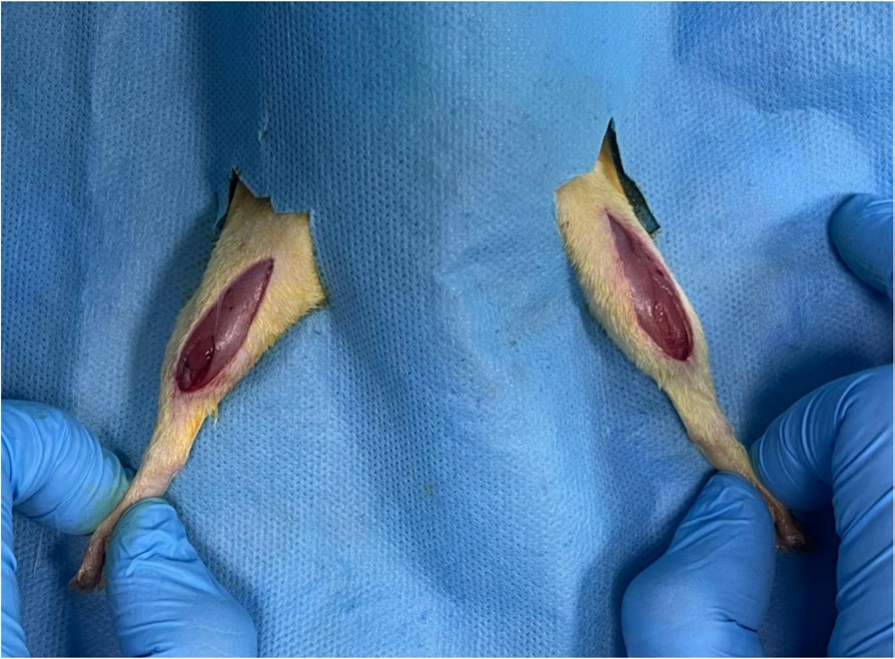
Bilateral hind limb fasciotomy of the rat

Anaesthesia was restored for the groups to undergo fasciatomy and cefazolin sodium was administered intraperitoneally as a single dose of 15 mg/kg to prevent surgical site infection. The right and left hip regions were then shaved and prepared with 10% povidone iodine. After sterile dressing, fasciotomy was performed by accessing the fascia through a 2–3 cm incision lateral and medial to the upper part of the hind limb (Fig. 3). After bleeding control and wound irrigation, the wound was closed with 3/0 absorbable sutures.

At the end of each week, rats in all groups were placed in a biological cage and urine samples were collected. At the end of the 4 th week, they were sacrificed and kidney tissue, muscle tissue and blood samples were collected.

### Biochemical ınvestigation

Creatinine kinase (CK), creatine kinase-myocardial band (CK-MB), blood urea nitrogen (BUN), creatine, alanine aminotransferase (ALT), aspartate aminotransferase (AST) were measured in rat blood serum samples centrifuged and stored at −20 C, Sodium (Na), phosphate (P), potassium (K), myoglobulin, calcium (Ca), lactate dehydrogenase (LDH) levels were measured.

In weekly urine samples, pH, urobilinogen, protein, erythrocytes, leucocytes, and specific gravity were measured.

### Histopathological examination

At the end of the study, crushed muscle and kidney tissue samples were taken from the sacrificed rats, fixed in 10% neutral buffered formalin solution, embedded in paraffin after automatic tissue tracking, 4 mm thick sections were cut from the paraffin blocks using a microtome (Leica, 2235), the sections were stained with haematoxylin–eosin, examined under a light microscope and the morphological changes observed were photographed. In the histopathological examination, the morphological changes in the muscle tissue in terms of regeneration, inflammation (cellular infiltration) and necrosis and the inflammatory cell infiltration in the kidney tissue were evaluated and photographed.

### Statistical analysis

In calculating the sample size for this study, the power for each variable was determined to be at least 80% with a type 1 error of 5%. The study used the Shapiro–Wilk test (n < 50) and non-parametric tests because the measurements were not normally distributed. Descriptive statistics for continuous variables in the study were expressed as'mean, standard deviation, median and range'. The Kruskal–Wallis H test was used to compare measurements between groups. Friedman's test was used to compare measurements between weeks, separately in the groups. Following the Kruskal–Wallis and Friedman tests, the'Post hoc test with Bonferroni correction'was used to determine different groups and weeks. Chi-square (Fisher's exact) test was calculated to determine the relationships between factors. The statistical significance level was set at 5% in the calculations and the SPSS statistical package programme (IBM SPSS for Windows, ver.26) was used for analysis.

## Results

All animals remained healthy throughout the study and were sacrificed at the end of the four-week observation period in accordance with the study protocol. No adverse events were observed during the study except for the complications mentioned in the Results section. Specifically, no wound site problems, infections or other post-operative complications were observed. All experimental procedures were performed within the required biosafety and ethical guidelines.

### Histopathological findings

#### Muscle tissue

**Fig. 4:**
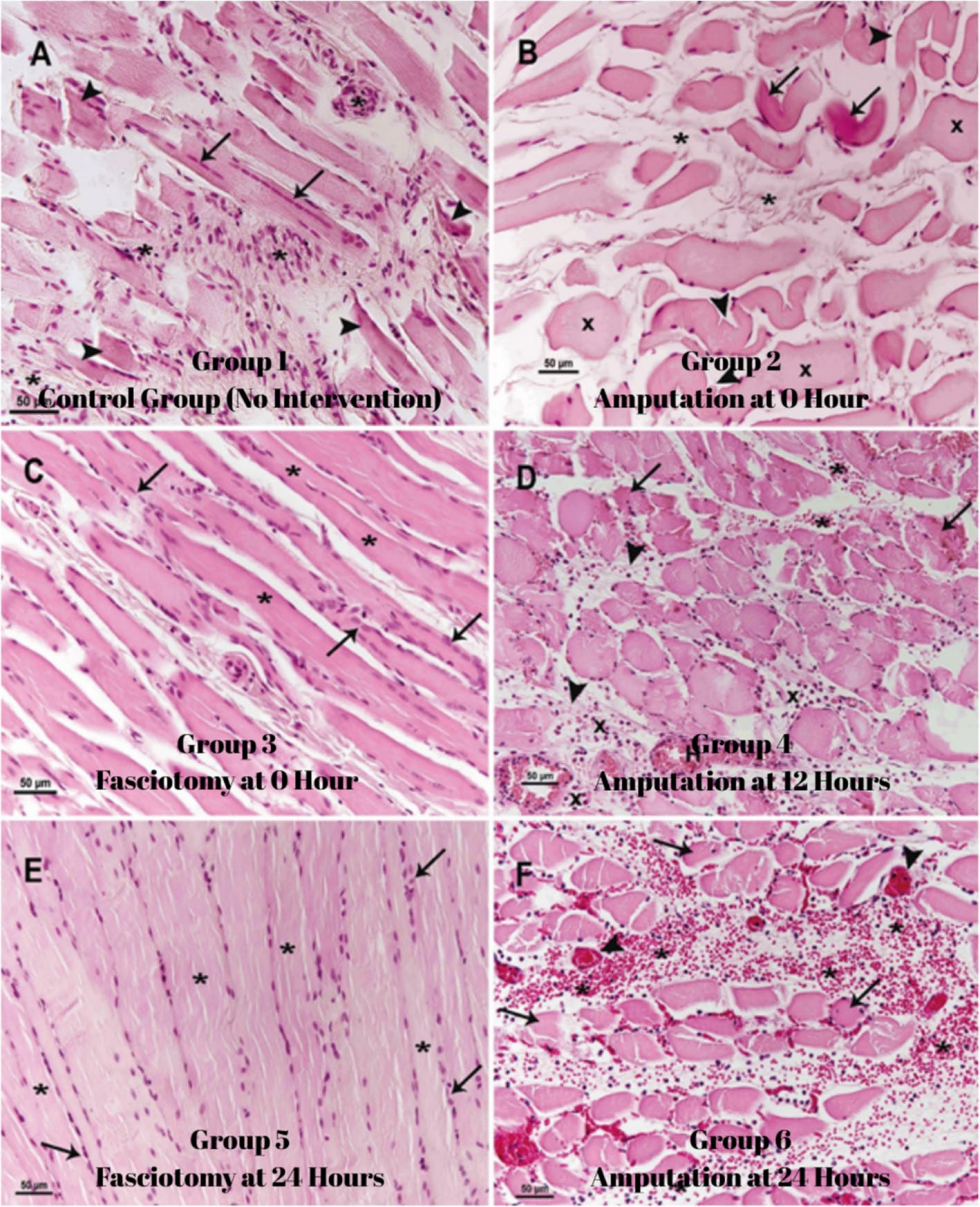
Group 1 (**A**) Muscle fibres have lost their regular and striated muscle structure and are necrotic (arrow), some muscle fibres are partially regenerated (arrows), mononuclear cell infiltrations and connective tissue cell proliferation (*) are observed between necrotic muscle fibres. Group 2 (**B**) muscle fibres are disrupted (arrowhead) and necrotic (arrow), muscle fibres are swollen (x) and interstitial oedema (*) is observed. Group 3 (**C**) muscle fibres are arranged in a regular structure (*) and regenerative muscle cell proliferation in muscle fibres (arrows). Group 4 (**D**) Muscle fibres lost their regular structure and fragmented necrosis (arrow), inflammatory cell infiltration between muscle fibres (x), haemorrhage (*) and fibrin threads (arrowhead), hyperaemia (H) and oedema in the interstitium. Group 5 (**E**) regular arrangement of muscle fibres (*) and muscle cell proliferation (arrow) indicating muscle fibre regeneration. Group 6 (**F**) shows disorganised and fragmented necrosis of muscle fibres (arrow), inflammatory cell infiltration and haemorrhage between muscle fibres (*), hyperaemia (arrow), fibrin threads and oedema

Group 1: In this group, sacrificed 4 weeks after rhabdomyolysis, irregularity, fragmentation and partial necrosis of muscle fibres were observed. Some muscle fibres showed partial regeneration, and mononuclear cell infiltration and connective tissue cell proliferation were observed in necrotic areas (Fig. 4A).

Group 2: In the group that underwent amputation immediately after rhabdomyolysis, muscle fibres were irregular, swollen and partially necrotic. Although hyperaemia and oedema were observed, there was no significant inflammatory cell infiltration in the interstitial tissue (Fig. 4B).

Group 3: In the group in which fasciotomy was performed immediately after rhabdomyolysis, muscle fibres showed a regular arrangement and signs of regeneration were detected. Hyperaemia and adipocyte infiltration were observed, but no significant necrotic changes were detected (Fig. 4C).

Group 4: In the group in which amputation was performed 12 h after rhabdomyolysis, the structure of the muscle fibres was disrupted and partial necrosis developed. Intense inflammatory cell infiltration, haemorrhage, fibrin threads, hyperemia and oedema were clearly observed in the interstitial space, indicating that the crush injury model had been successfully created (Fig. 4D).

Group 5: In the group in which fasciotomy was performed 24 h after rhabdomyolysis, muscle fibres retained their regular arrangement and striated muscle structure. There was no inflammatory cell infiltration except for muscle cell proliferation, indicating regeneration (Fig. 4E).

Group 6: In the group that underwent amputation 24 h after rhabdomyolysis, muscle fibres were disintegrated, necrotic and lost their striated structure. Intense inflammatory cell infiltration, fibrin threads, capillary hyperemia, haemorrhage and interfibrillar oedema were more prominent compared to group 3 (Fig. 4F).

#### Kidney tissue

In rats sacrificed four weeks after rhabdomyolysis (Group 1), mononuclear cell infiltration (multifocal interstitial nephritis) was observed in the intertubular, perivascular, and periglomerular regions. In contrast, no significant cellular infiltration was found in the group that underwent immediate amputation (Group 2). Furthermore, inflammatory infiltration was significantly milder in the other groups compared to the control group. The differences between these groups are outlined in Table 1 [17, 18].

**Table 1:** Chronic effects of rhabdomyolysis on renal tissues

	**Group 1**	**Group 2**	**Group 3**	**Group 4**	**Group 5**	**Group 6**	*p*-value^*^
Mild	1	0	3	2	2	2	,032
Moderate	1	0	3	2	4	4	
Severe	6	0	0	0	2	0	

^*^Significance level according to the results of chi-square (Fisher's exact) test

**Fig. 5:**
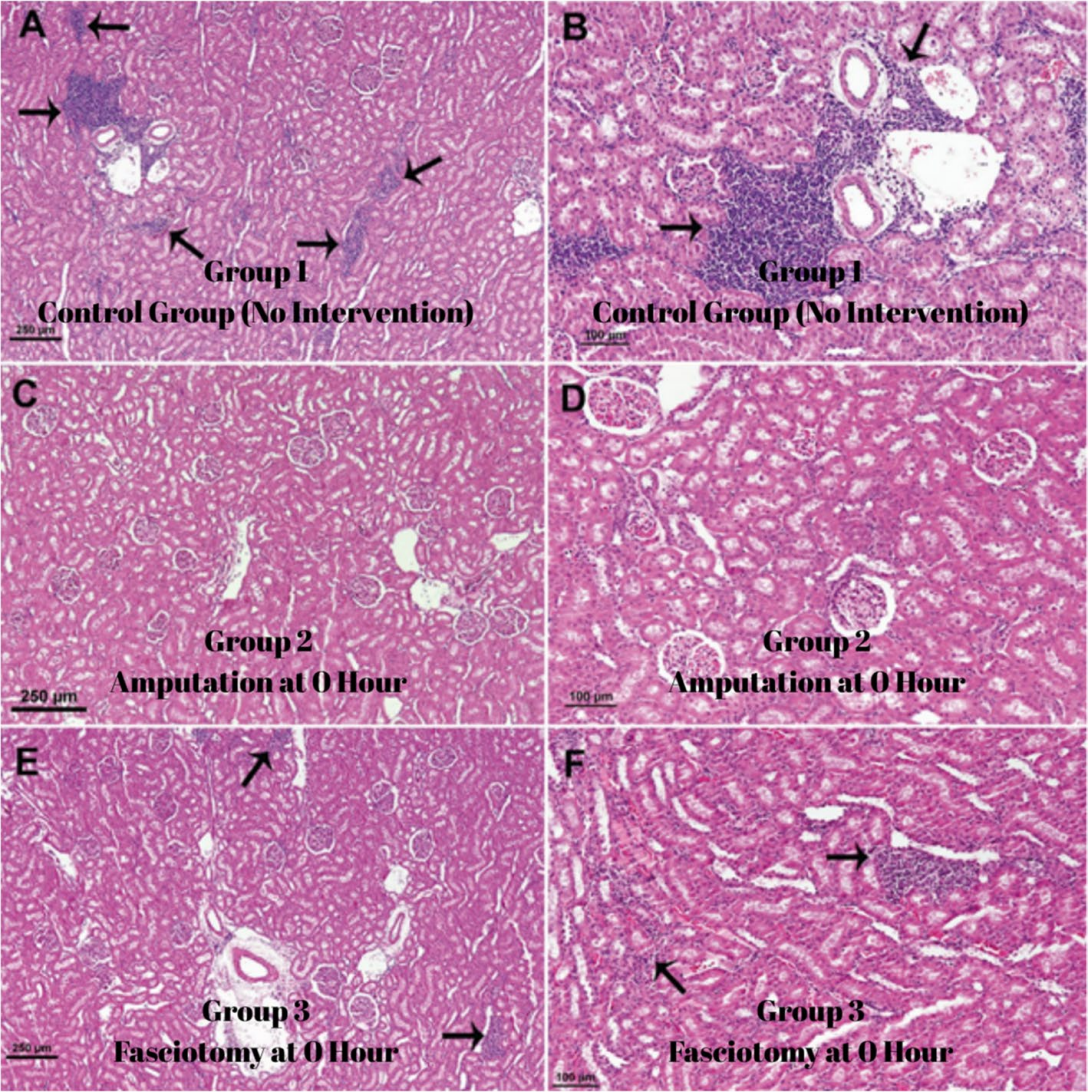
Note that mononuclear cell infiltrations (arrows) in the renal interstitium are prominent in figures **A**-**B** (Group 1), less prominent in figures **E**–**F** (Group 3) and absent in figures **C**-**D** (Group 2)

**Fig. 6:**
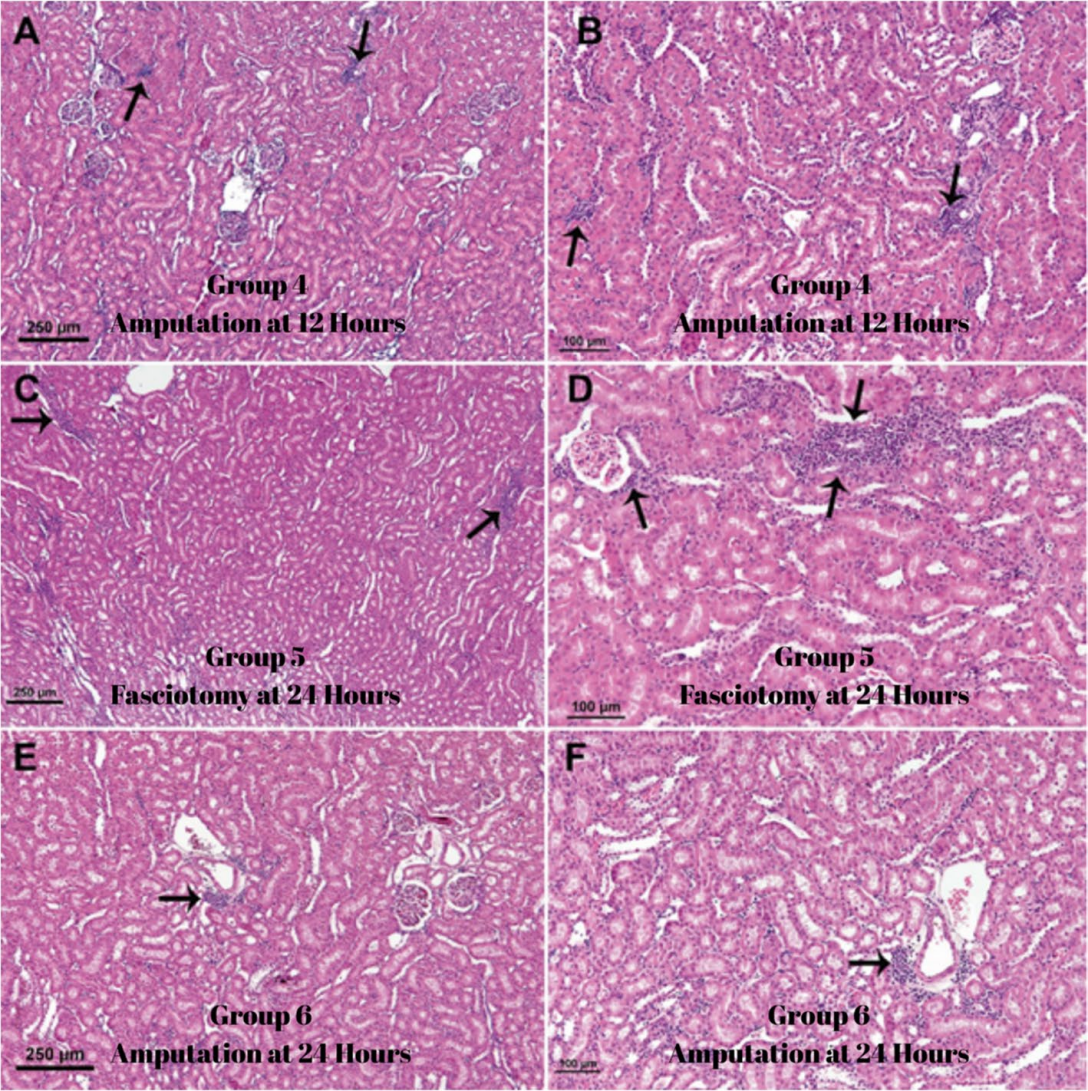
**A**-**B** (Group 4), **C**-**D** (Group 5) and **E**–**F** (Group 6) figures show mononuclear cell infiltrations (arrows) in the interstitium

In the group sacrificed 4 weeks after rhabdomyolysis (group 1), the most basic morphological change on microscopic examination of the kidneys was the presence of non-purulent multifocal interstitial nephritis foci in the interstitium. However, it was noteworthy that these interstitial nephritis foci were not observed in the kidneys of rats that underwent amputation immediately after rhabdomyolysis (group 2), whereas they were observed in the kidneys of rats that underwent amputation or fasciotomy later after rhabdomyolysis (groups 3,4,5 and 6), although significantly less than in the control group (group 1) (Figs. 5 and 6). As all the kidneys examined in the study were sacrificed and examined 4 weeks after rhabdomyolysis, no signs of acute tubular damage were observed in the kidneys; these damages healed with regeneration within 4 weeks and only mononuclear cell infiltrations, characteristic of chronic inflammation, were observed in the interstitium. In fact, although degenerative necrotic changes were observed in the muscle tissue in the group (group 2) that was amputated immediately after rhabdomyolysis, no neutrophil leukocyte infiltrations were observed.

### Biochemical Findings

As a result of the study, statistically significant differences were observed between the groups in various biochemical parameters. A significant difference was found between the groups for ALT levels (*p* = 0.016), and in particular the difference between the control group and group 4 was statistically significant (*p* < 0.05). This significance has no clinical value. The other groups were similar to the control group.

A statistically significant difference was found between the groups in AST level (*p* = 0.024), especially between the control group and the 6 th group (*p* < 0.05). Although a significant difference was observed in the Ca level (*p* = 0.026), when comparing the pairs between the groups, no significant difference was found with the control group (*p* > 0.05). A statistically significant difference was also observed between the groups in the CK level (*p* = 0.035). In particular, the difference between the control group and the 6 th group was found to be significant (*p* < 0.05). A significant difference was observed at the level of CKMB (*p* = 0.016), especially between the control group and the 5 th and 6 th groups (*p* < 0.05). As a result of the analyses performed for creatine, a significant difference was found between the control group and the 2nd and 4 th groups (*p* < 0.05). There was no significant difference between the control group and the 6 th group (*p* > 0.05) There was no statistically significant difference between the control group and groups 3 and 5 (*p* > 0.05). Significant differences were observed between the control group and groups 2, 4 and 6 (*p* < 0.05). Significant differences were observed between the control group and the 4 th group for K (*p* < 0.05) and between the control group and the 3rd, 4 th and 5 th groups for Na (*p* < 0.05). No significant difference was found between the groups at the P level (*p* = 0.460) (Table 2).

**Table 2:** Serum biochemical parameters of each group at the 4 th week after rhabdomyolysis

Parameter	Group 1 (Mean ± SD)	Group 2 (Mean ± SD)	Group 3 (Mean ± SD)	Group 4 (Mean ± SD)	Group 5 (Mean ± SD)	Group 6 (Mean ± SD)	*p*-value*
ALT (U/L)	33.00 ± 4.86a	29.60 ± 4.43ab	33.61 ± 4.04a	26.68 ± 2.81b	31.58 ± 3.99a	30.90 ± 3.01a	0.016
AST (U/L)	82.07 ± 11.64a	85.71 ± 8.29a	82.63 ± 10.66a	74.35 ± 10.87ab	69.55 ± 8.30ab	76.97 ± 13.80b	0.024
CK (U/L)	525.25 ± 125.72a	598.38 ± 110.09ab	448.75 ± 106.40ab	514.75 ± 216.63ab	418.88 ± 100.45ab	375.13 ± 245.80b	0.035
CKMB (U/L)	956.43 ± 234.05a	1089.88 ± 199.01ab	775.14 ± 183.23ab	917.91 ± 384.64ab	754.36 ± 174.05b	657.27 ± 443.90b	0.016
LDH (U/L)	903.00 ± 228.27ab	1036.38 ± 197.46a	732.25 ± 186.01ab	851.62 ± 334.88ab	608.00 ± 240.14b	649.63 ± 454.42b	0.015
CRE (mg/dL)	0.28 ± 0.04a	0.23 ± 0.04b	0.30 ± 0.04a	0.23 ± 0.03b	0.28 ± 0.05a	0.29 ± 0.02a	0.004
URE (mg/dL)	47.01 ± 3.69a	43.33 ± 2.94b	49.14 ± 5.21a	41.75 ± 3.68b	49.41 ± 3.19a	43.58 ± 3.13b	0.001
Na (mEq/L)	144.13 ± 12.30a	141.13 ± 29.26a	134.88 ± 3.60b	132.63 ± 2.67b	134.50 ± 1.93b	145.87 ± 14.35a	0.008
K (mEq/L)	5.61 ± 1.05a	5.41 ± 1.05ab	5.11 ± 0.38ab	4.85 ± 0.09b	5.29 ± 0.22ab	5.20 ± 0.47ab	0.037
Ca (mg/dL)	9.23 ± 0.47ab	8.97 ± 0.51ab	9.42 ± 0.42ab	8.88 ± 0.35b	9.57 ± 0.49a	9.48 ± 0.36ab	0.026
P (mg/dL)	6.38 ± 0.55	6.02 ± 0.33	6.73 ± 0.87	6.51 ± 1.12	6.65 ± 1.22	6.48 ± 0.46	0.460

*SD* Standart Deviation, *ALT* Alanine Aminotransferase, *AST* Aspartate Aminotransferase, *Ca* Calcium, *CK* Creatine Kinase, *CK-MB* Creatine Kinase-Muscle Brain, *CRE* Creatinine, *K* Potassium, *LDH* Lactate Dehydrogenase, *Na* Sodium, *P* Phosphorus, *URE* Urea

^a,b^Indicates the difference between paired groups according to Bonferroni corrected Post-Hoc test

^*^Significance level between groups according to Kruskal–Wallis H Test

### Weekly changes ın urine biochemical parameters

#### First week results

**Fig. 7:**
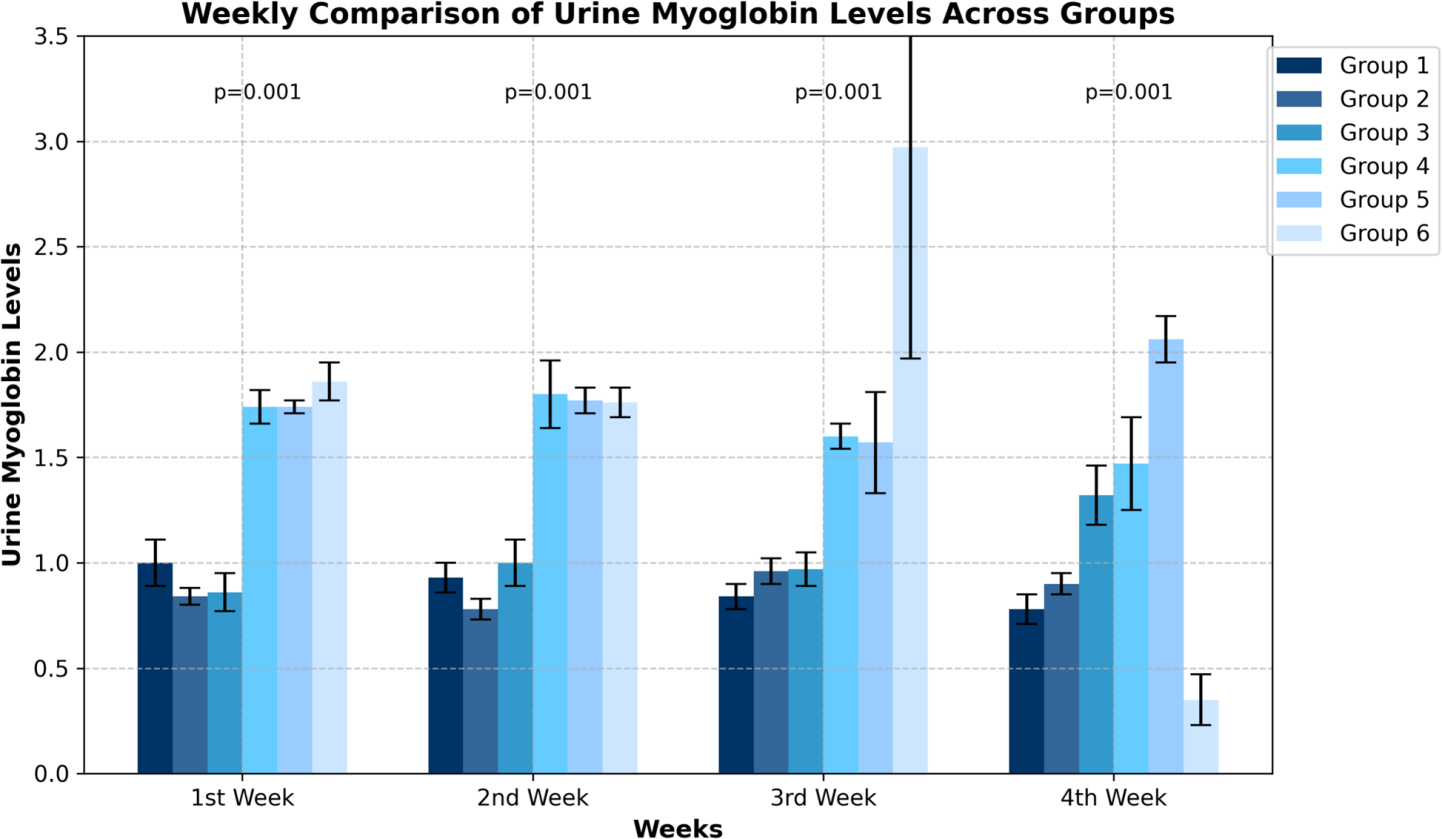
Urine myoglobin levels of the groups and weekly comparison results

Urinary myoglobin levels showed a significant difference between the groups according to the treatment method (*p* = 0.001). Compared with the control group:

Groups 2 and 3 (early fasciotomy and amputation groups): Urine myoglobin levels were significantly lower. Groups 4, 5 and 6: Urine myoglobin levels were significantly higher (Fig. 7).

#### Second week results

Significant differences in urine myoglobin levels were observed between groups in week 2 (*p* = 0.001). The differences in this week were particularly observed between the groups that started treatment early (groups 2 and 3) and the groups that delayed treatment (groups 4, 5 and 6). During the second week, urinary myoglobin levels remained relatively lower in the early treatment groups.

#### Third week results

During the third week, the differences between the groups became more pronounced (*p* = 0.001). During this week.

Control group: A time-dependent decrease in urinary myoglobin levels was observed.

Groups 2 and 3: In these groups, where fasciotomy and amputation were performed at the time of injury, urinary myoglobin levels remained low.

Groups 4, 5 and 6: In the groups where treatment was delayed, urinary myoglobin levels remained high. In particular, in groups 5 and 6, a higher and more variable course was observed compared to early treatment.

#### Fourth week results

In the fourth week analyses, the differences between the groups remained significant (*p* = 0.001):

Group 4: Urinary myoglobin levels showed a decreasing trend from this week onwards.

Group 5 and Group 6: Urinary myoglobin levels remained fluctuatingly high in these groups where fasciotomy and amputation were not performed early (Table 3).

**Table 3:** Urine myoglobin levels of the groups and weekly comparison results

Week	Group 1 (Mean ± SD)	Group 2 (Mean ± SD)	Group 3 (Mean ± SD)	Group 4 (Mean ± SD)	Group 5 (Mean ± SD)	Group 6 (Mean ± SD)	*p-value*
1 st Week	A1.00 ± 0.11 (c)	B0.84 ± 0.04 (d)	C0.86 ± 0.09 (d)	A1.74 ± 0.08 (b)	B1.74 ± 0.03 (b)	B1.86 ± 0.09 (a)	0.001*
2nd Week	A0.93 ± 0.07 (b)	C0.78 ± 0.05 (c)	A1.00 ± 0.11 (b)	A1.80 ± 0.16 (a)	B1.77 ± 0.06 (a)	C1.76 ± 0.07 (a)	0.001*
3rd Week	B0.84 ± 0.06 (d)	A0.96 ± 0.06 (c)	A0.97 ± 0.08 (c)	B1.60 ± 0.06 (b)	C1.57 ± 0.24 (b)	A2.97 ± 1.00 (a)	0.001*
4 th Week	B0.78 ± 0.07 (c)	A0.90 ± 0.05 (c)	B1.32 ± 0.14 (b)	B1.47 ± 0.22 (b)	A2.06 ± 0.11 (a)	D0.35 ± 0.12 (d)	0.001*
^**^p	***,001***	***,001***	***,001***	***,004***	***,001***	***,001***	

*SD* Standard Deviation, *p*-value Statistical significance between groups

^*^Significance level between groups according to Kruskal–Wallis H Test □ a,b,c: Indicates the difference between paired groups according to Bonferroni corrected Post-Hoc test

^**^Significance levels between measurement periods according to Friedman test result □ A,B,C: Shows the difference between measurement periods according to Bonferroni corrected Post-Hoc test

**Fig. 8:**
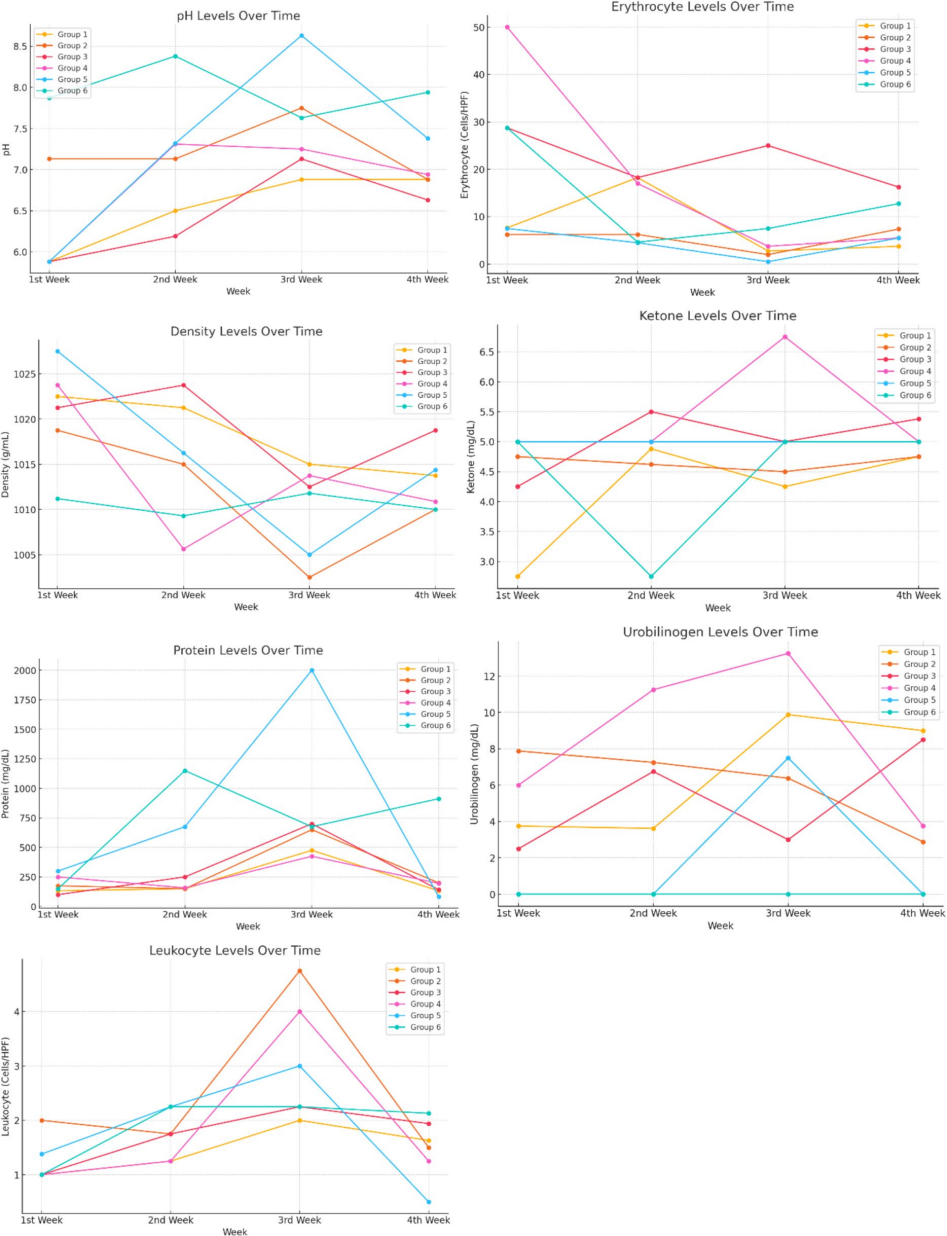
Weekly changes in urine biochemical parameters

In the present study, the alterations in urine analysis parameters (leukocytes, urobilinogen, protein, pH, erythrocytes, density and ketones) over time in diverse groups were examined. The findings demonstrated that these parameters exhibited variations depending on group structure and temporal factors (Table 4). Leukocyte levels exhibited significant fluctuations over time in all groups except group 6. Time-dependent changes in urobilinogen levels were observed in groups 2, 4 and 5, though these changes were not deemed to be of clinical significance. Group 2, 3, 4, 5 and 6 demonstrated differences in protein levels and pH values when compared to the control group. Urine protein levels showed more elevations and fluctuations in groups 5 and 6, suggesting that delayed treatment may affect renal function. Erythrocyte levels, which were significant in the initial weeks, demonstrated temporal changes. Urine density exhibited time-dependent changes in groups 2, 3, 4 and 5. Significant increases in ketone levels were observed in the first weeks compared to the control group, though these were not deemed to be clinically significant. The study concluded that while the parameters exhibited significant variations over time, these changes were not deemed to be of clinical significance. In summary, the urinary parameters demonstrated variability influenced by group structure and temporal factors. However, these variations were not considered to be of clinical importance (Fig. 8).

**Table 4:** Weekly urine parameters of the groups

		**Group 1**	**Group 2**	**Group 3**	**Group 4**	**Group 5**	**Group 6**	*p-value
		Mean ± SD	Mean ± SD	Mean ± SD	Mean ± SD	Mean ± SD	Mean ± SD	
**Urobilinogen**	1 st Week	B1.00 ± 0.00 (b)	B2.00 ± 0.76 (a)	C1.00 ± 0.00 (b)	B1.00 ± 0.00 (b)	B1.38 ± 0.44 (b)	B1.00 ± 0.76 (b)	0.001
	2nd Week	B1.25 ± 0.46 (b)	B1.75 ± 0.71 (b)	B1.75 ± 0.71 (b)	B1.25 ± 0.46 (b)	A2.25 ± 1.16 (a)	A2.25 ± 1.16 (a)	0.060
	3rd Week	A2.00 ± 1.07 (c)	A4.75 ± 2.55 (a)	A2.25 ± 1.28 (c)	A4.00 ± 2.62 (a)	A3.00 ± 1.07 (b)	A2.25 ± 1.13 (c)	0.016
	4 th Week	B1.63 ± 0.52 (a)	B1.50 ± 0.53 (a)	B1.94 ± 1.32 (a)	B1.25 ± 0.46 (a)	B0.50 ± 0.53 (b)	A2.13 ± 1.25 (a)	0.008
^**^p-value		_<*0,05*_	_<*0,05*_	_>*0,05*_	_<*0,05*_	_<*0,05*_	_>*0,05*_	
**Leukocyte**	1 st Week	B3.75 ± 2.55 (b)	A7.88 ± 2.10 (a)	B2.50 ± 0.53 (b)	B6.00 ± 4.00 (a)	B0.00 ± 0.00 (c)	A0.00 ± 0.00 (c)	0.001
	2nd Week	B3.62 ± 1.06 (c)	A7.25 ± 1.67 (b)	A6.75 ± 3.24 (b)	A11.25 ± 2.31 (a)	B0.00 ± 0.00 (d)	A0.00 ± 0.00 (d)	0.001
	3rd Week	A9.88 ± 3.00 (b)	A6.38 ± 1.30 (b)	B3.00 ± 3.30 (c)	A13.25 ± 1.98 (a)	A7.50 ± 8.02 (b)	A0.00 ± 0.00 (d)	0.001
	4 th Week	A9.00 ± 1.20 (a)	A2.88 ± 1.13 (b)	A8.50 ± 1.60 (a)	B3.75 ± 6.94 (b)	B0.00 ± 0.00 (c)	A0.00 ± 0.00 (c)	0.001
**p		_<*0,05*_	_<*0,05*_	_<*0,05*_	_<*0,05*_	_<*0,05*_	_>*0,05*_	
**Protein**	1 st Week	C132.50 ± 107.80(c)	B175.00 ± 103.51(b)	C100.00 ± 0.00 (c)	B250.00 ± 92.58(a)	B300.00 ± 0.00 (a)	C150.00 ± 92.58(c)	0.001
	2nd Week	B150.00 ± 92.58(b)	B150.00 ± 92.58 (b)	B250.00 ± 92.58(b)	C157.50 ± 121.51(b)	A675.00 ± 822.45(a)	A1150.00 ± 908.69a	0.001
	3rd Week	A475.00 ± 324.04(b)	A650.00 ± 374.17(b)	A700.00 ± 434.25(b)	A425.00 ± 365.47(b)	B2000.00 ± 0.00 (a)	B675.00 ± 822.45(b)	0.001
	4 th Week	C132.50 ± 107.80(b)	B200.00 ± 106.90(b)	C141.25 ± 100.92(b)	B195.00 ± 326.80(b)	C82.50 ± 32.40 (b)	A912.50 ± 903.07(a)	0.001
^**^p		_<*0,05*_	_<*0,05*_	_<*0,05*_	_<*0,05*_	_<*0,05*_	_<*0,05*_	
**pH**	1 st Week	B5.88 ± 0.23 (c)	B7.13 ± 0.44 (b)	B5.88 ± 0.35 (c)	B5.88 ± 0.23 (c)	B5.88 ± 0.23 (ac)	B7.87 ± 0.95 (a)	0.001
	2nd Week	A6.50 ± 0.53 (c)	B7.13 ± 0.44 (b)	B6.19 ± 0.26 (d)	A7.31 ± 0.37 (b)	A7.32 ± 1.08 (b)	A8.38 ± 0.69 (a)	0.001
	3rd Week	A6.88 ± 0.69 (b)	B7.75 ± 0.27 (b)	A7.13 ± 1.22 (b)	A7.25 ± 0.80 (b)	A8.63 ± 0.52 (a)	B7.63 ± 1.48 (b)	0.008
	4 th Week	A6.88 ± 0.44 (b)	A6.88 ± 0.23 (b)	B6.63 ± 1.03 (b)	B6.94 ± 0.42 (b)	A7.38 ± 0.88 (a)	B7.94 ± 1.08 (a)	0.016
^**^p		_<*0,05*_	_>*0,05*_	_<*0,05*_	_<*0,05*_	_<*0,05*_	_>*0,05*_	
**Ketone**	1 st Week	B2.75 ± 0.89 (c)	A4.75 ± 0.46 (a)	B4.25 ± 0.89 (b)	B5.00 ± 0.00 (a)	A5.00 ± 0.00 (a)	A5.00 ± 0.00 (a)	0.001
	2nd Week	4 A.88 ± 0.35 (a)	A4.62 ± 0.52 (a)	A5.50 ± 0.93 (a)	B5.00 ± 0.00 (a)	A5.00 ± 0.00 (a)	B2.75 ± 2.00 (b)	0.001
	3rd Week	A4.25 ± 0.46 (b)	A4.50 ± 0.53 (b)	A5.00 ± 0.00 (b)	A6.75 ± 1.73 (a)	A5.00 ± 0.00 (b)	A5.00 ± 0.00 (b)	0.001
	4 th Week	A4.75 ± 0.46 (b)	A4.75 ± 0.46 (b)	A5.38 ± 0.52 (a)	B5.00 ± 0.00 (b)	A5.00 ± 0.00 (b)	A5.00 ± 0.00 (b)	0.008
^**^p		_<*0,05*_	_>*0,05*_	_<*0,05*_	_<*0,05*_	_>*0,05*_	_<*0,05*_	
**Density**	1 st Week	A1022.50 ± 2.67 (a)	A1018.75 ± 5.82 (b)	A1021.25 ± 3.54 (a)	A1023.75 ± 2.31 (a)	A1027.50 ± 2.67 (a)	A1011.20 ± 9.54 (b)	0.001
	2nd Week	A1021.25 ± 2.31 (a)	A1015.00 ± 5.35 (b)	A1023.75 ± 2.31 (a)	C1005.63 ± 4.17 (d)	A1016.25 ± 10.26(b)	A1009.30 ± 9.04 (c)	0.001
	3rd Week	B1015.00 ± 10.69(b)	B1002.50 ± 2.67 (b)	B1012.50 ± 13.36(b)	B1013.75 ± 10.26(b)	B1005.00 ± 9.26 (b)	A1011.80 ± 12.80(b)	0.114
	4 th Week	B1013.75 ± 7.91 (b)	A1010.00 ± 7.56 (b)	B1018.75 ± 12.17(b)	B1010.88 ± 6.36 (b)	A1014.38 ± 9.04 (a)	A1010.00 ± 8.45 (b)	0.322
^**^p		_>*0,05*_	_<*0,05*_	_<*0,05*_	_<*0,05*_	_<*0,05*_	_>*0,05*_	
**Erythrocyte**	1 st Week	B7.63 ± 0.52 (c)	A6.25 ± 2.05 (c)	A28.75 ± 22.72 (b)	A50.00 ± 0.00 (a)	A7.50 ± 0.53 (c)	A28.75 ± 22.72 (b)	0.001
	2nd Week	A18.25 ± 19.60 (a)	A6.25 ± 2.05 (b)	B18.25 ± 19.60 (a)	B17.00 ± 20.47 (a)	A4.50 ± 3.42 (b)	D4.63 ± 3.85 (b)	0.109
	3rd Week	C2.75 ± 2.87 (b)	B2.00 ± 3.12 (b)	A25.00 ± 26.73 (a)	C3.75 ± 4.03 (b)	C0.50 ± 0.53 (b)	C7.50 ± 0.53 (b)	0.001
	4 th Week	C3.75 ± 2.76 (b)	A7.38 ± 1.06 (a)	B16.25 ± 21.09 (b)	C5.50 ± 3.42 (b)	A5.50 ± 3.42 (b)	B12.75 ± 15.06 (b)	0.172
^**^p		_<*0,05*_	_<*0,05*_	_<*0,05*_	_<*0,05*_	_>*0,05*_	_<*0,05*_	

*SD* Standard Deviation, *Med* Median, *p*-value Statistical significance between groups

^*^Significance level between groups according to Kruskal–Wallis H Test

^a,b,c^Indicates the difference between paired groups according to Bonferroni corrected Post-Hoc test

^**^Significance levels between measurement periods according to Friedman test result

^A,B,C^Shows the difference between measurement periods according to Bonferroni corrected Post-Hoc test

## Discussion

The present study demonstrates that the timing of fasciotomy and amputation after rhabdomyolysis is crucial for reducing tissue damage and preventing systemic complications. Early intervention, particularly early amputation, was found to better limit inflammation and degeneration in kidney tissue, preserving kidney function and preventing crush syndrome more effectively than fasciotomy when both lower limbs were exposed to prolonged elevated pressures.

Acute kidney injury (AKI), one of the most lethal complications of CS, has been observed in a significant proportion of patients diagnosed with CS [19, 20]. A study conducted after the Wenchuan earthquake revealed that AKI developed in 41.6% of patients [21]. The extant literature on the subject is somewhat limited in scope, and thus far there has been no consensus regarding which patients require surgical intervention, when the intervention should be performed, and the optimal method for performing the intervention in the treatment of CS [22]. A paucity of knowledge and experience regarding disaster preparedness and the surgical treatment of crush injuries and CS is evident within the medical community [5, 7, 23, 24].

The surgical treatment options that are currently under debate, such as fasciotomy and amputation, as well as the optimal timing of these procedures, are the subject of considerable controversy [8, 25–27]. The primary objective of fasciotomy is to reverse muscle and tissue damage. However, the probability of success is reduced as the time elapsed after injury increases. Furthermore, there are secondary disadvantages associated with fasciotomy, such as infection and fluid loss [25].

A plethora of divergent opinions have been posited within the extant literature when comparing patients who underwent fasciotomy with those who did not [28, 29]. Matsuoka et al. found no evidence that fasciotomy improved outcomes in crush injury patients [28]. Additionally, Sever et al. noted that fasciotomies performed in chaotic conditions with multiple injuries could become complicated due to inadequate wound care and increased risks. They therefore emphasised that fasciotomies should not be recommended as a routine procedure [30]. Conversely, an alternative viewpoint asserts that fasciotomy performed at the time of presentation following crush injury offers a potential opportunity to reverse tissue damage [31].

It is postulated that delayed fasciotomy may result in an increased risk of infection and sepsis. In a study by Duman et al., 25% of cases reported resistant infections following fasciotomy, with these cases necessitating amputation [23]. Furthermore, field experiences frequently reported in various disaster areas indicate that infection and associated amputation rates are high when fasciotomy is performed, regardless of conditions in the presence of CS [9, 32, 33]. Sever et al. reported a 24.8% sepsis rate and a 16.4% mortality rate following fasciotomy. These rates are higher than those observed in cases where fasciotomy was not performed [4].

Zhang et al. reported that 33% of amputations were due to infection following delayed or incomplete fasciotomy [34]. The prevailing opinion among experts in the field is that fasciotomy carries a significant risk of infection. It has also been reported that the sequelae of infection are much worse than late muscle contractures caused by muscle fibrosis and do not contribute to long-term functional recovery of the muscle [28, 35]. Fasciotomy, a surgical procedure performed on limbs with dead muscle tissue, has been shown to increase reperfusion and metabolic products from necrotic tissue. This post-ischaemic reperfusion has been demonstrated to be an effective mechanism in the development of CS [36].

In the present study, a statistically significant difference was identified between the groups with respect to AST levels. However, the disparity observed between Group 1 (82.07 ± 11.64 U/L) and Group 6 (76.97 ± 13.80 U/L) was not deemed to be of clinical significance. The values of both groups are within the reference range, and this discrepancy does not result in a clinical outcome.

The presence of irregularities, necrosis, intense inflammation, fibrin strands, hyperemia, bleeding, and oedema in muscle fibres in the groups amputated at 12 and 24 h indicates that a successful crush injury model was established in rats. At the conclusion of the study, morphological changes were found to be milder in the fasciotomy and amputation groups in comparison to the control group. Muscle fibres were found to be regular, and regeneration was evident in the fasciotomy groups. In the immediate amputation group, necrosis and oedema were observed, while bleeding and inflammation were not detected; in the delayed amputation groups, significant hyperemia, bleeding, oedema, and widespread inflammation were detected.

In the context of natural disasters, limb amputation has emerged as a prevalent, intricate, and contentious concern, particularly in the aftermath of earthquakes [7, 13, 37]. Some medical practitioners advocate for early amputation as a preventative measure against life-threatening complications of CS and to mitigate the risk of infection [4]. Matsuoka et al. [8, 28] state that amputation is the ultimate treatment option in cases of CS, and is indicated in cases of life-threatening infection or necrosis. However, the findings of this study suggest that emergency amputation may be more beneficial than delayed amputation. This result is consistent with the findings of Kundakçı et al., who reported that amputation may reduce the need for dialysis. The significance of prompt and transformative surgical interventions is accentuated in mass injury scenarios, such as earthquakes, where access to dialysis is constrained [6].

Kidney tissue analysis has demonstrated that early amputation prevents acute tubular damage, while delayed amputation worsens the outcome. Early intervention has been shown to maintain low and stable urine myoglobin levels, while delayed intervention has been associated with elevated and variable levels. In addition, the findings of Koyuncu et al. support the hypothesis that early amputation has a beneficial effect on kidney function, as indicated by higher dialysis requirements and complication rates in patients who underwent fasciotomy, as reported in this study [38].

In conclusion, the present study demonstrates that the timing of surgical intervention is critical in CS. In the experimental model utilised, early amputation proved to be a more efficacious approach in comparison to early fasciotomy. The timing of surgical intervention in CS is of vital importance, and early amputation should be considered a strong option to minimise systemic effects. However, the present study was conducted in an experimental rat model, and further clinical studies are required to determine the applicability of these findings to humans. In order to replicate similar results in humans, large-scale clinical studies and multidisciplinary evaluations are necessary. Consequently, it is imperative to methodically assess decisions pertaining to early amputation within a clinical context.

### Limitations

One limitation of our study is that blood biochemical parameters were not monitored hourly because it is very difficult to obtain tail blood hourly in practice. Another limitation was that we followed the rats as groups in cages, so daily intake and output could not be monitored. Another limitation is that, due to the extremely thin and flexible nature of the fascia layer in rats, it has been documented that the histopathological findings obtained in this model do not fully correspond to the compartment syndrome observed in humans following crush injuries [39]. This suggests that the translational value of the rat model is limited.

## Supplementary Information

Supplementary Material 1.

## Data Availability

Data provided in manuscript files.

## Abbreviations

CS: Crush syndrome
CK: Creatinine kinase
CK-MB: Creatine kinase-myocardial band
BUN: Blood urea nitrogen
ALT: Alanine aminotransferase
AST: Aspartate aminotransferase (serum samples centrifuged and stored at -20 C
Na: Sodium
P: Phosphate
K: Potassium oglobulin
Ca: Calcium
LDH: Lactate dehydrogenase
AKI: Acute kidney injury
