# Supplementary material for: Experimental evaluation of timing and preference of surgical ıntervention for crush syndrome in disaster scenarios: fasciotomy or amputation? A rat model study
Source: J Orthop Surg Res. 2025 May 22;20:500. doi: 10.1186/s13018-025-05927-5 (PMC12100787; doi:10.1186/s13018-025-05927-5)
Supplement: Supplementary file 1 — Supplementary Material 1. [file 13018_2025_5927_MOESM1_ESM.docx]

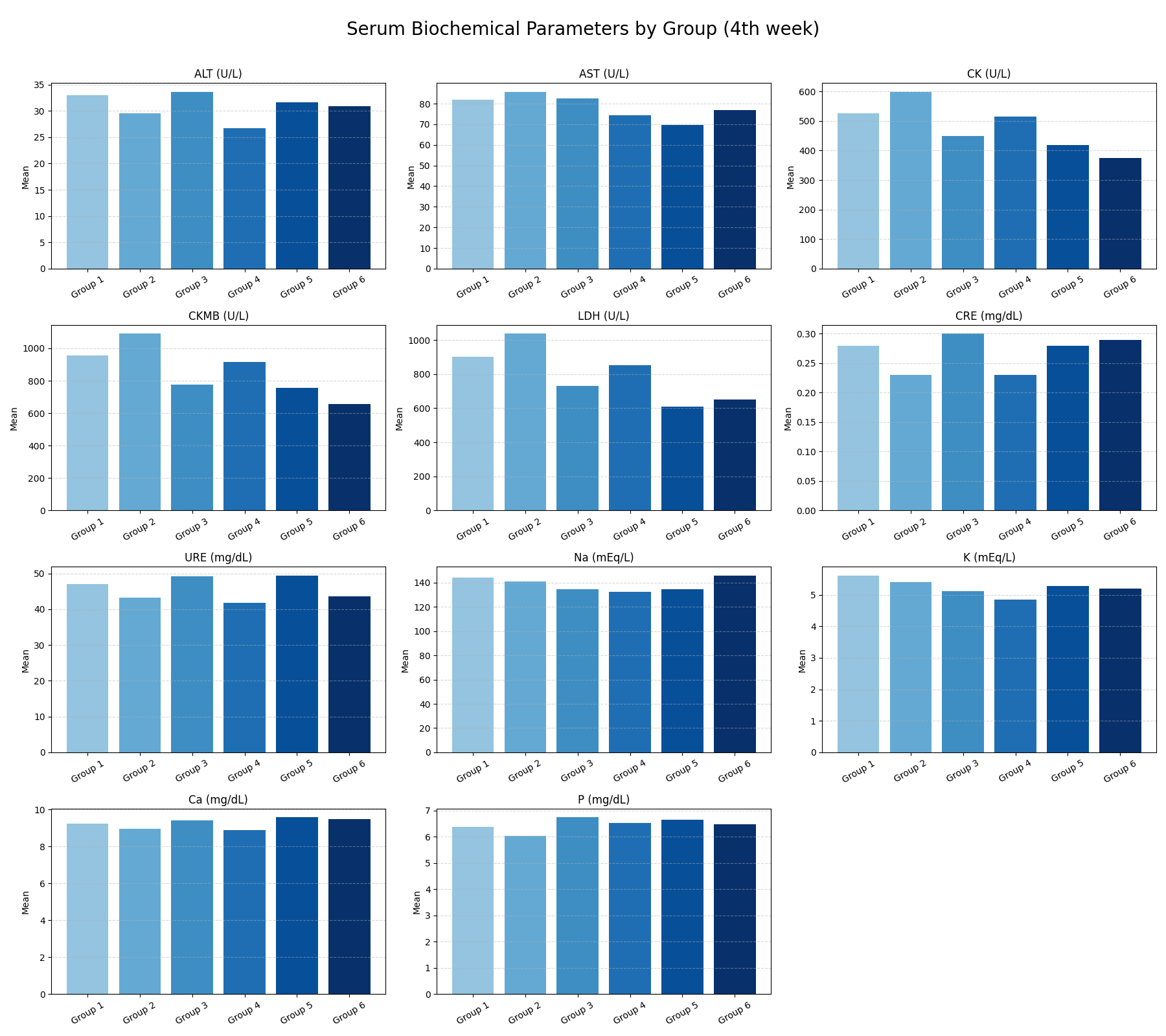


*ALT: Alanine Aminotransferase, AST: Aspartate Aminotransferase, Ca: Calcium, CK: Creatine Kinase, CK-MB: Creatine Kinase-Muscle Brain, CRE: Creatinine, K: Potassium, LDH: Lactate Dehydrogenase, Na: Sodium, P: Phosphorus, URE: Urea*

Figure. Serum biochemical parameters of each group at the 4th week after rhabdomyolysis.
